# Utility of photon-counting detectors for MV-kV dual-energy computed tomography imaging

**DOI:** 10.1117/1.JMI.11.S1.S12811

**Published:** 2024-12-26

**Authors:** Giavanna Jadick, Maya Ventura, Patrick J. La Rivière

**Affiliations:** a University of Chicago, Department of Radiology, Chicago, Illinois, United States; b University of Chicago Medical Center, Comprehensive Cancer Center, Chicago, Illinois, United States

**Keywords:** computed tomography, dual energy, estimation theory, megavoltage imaging, photon-counting detector, simulation

## Abstract

**Purpose:**

High soft-tissue contrast imaging is essential for effective radiotherapy treatment. This could potentially be realized using both megavoltage and kilovoltage x-ray sources available on some therapy treatment systems to perform “MV-kV” dual-energy (DE) computed tomography (CT). However, noisy megavoltage images obtained with existing energy-integrating detectors (EIDs) are a limiting factor for clinical translation. We explore the potential for non-spectral photon-counting detectors (PCDs) to improve MV-kV image quality simply by equally weighting all MV photons rather than up-weighting the less informative, lower contrast high-energy photons as in an EID.

**Approach:**

Three computational methods were applied to compare non-spectral PCDs with EIDs in MV-kV DE imaging. A single-line integral estimation theory approach was used to calculate the basis material signal-to-noise ratio (SNR) of tissue (1 to 50 cm) and bone (0.1 to 10 cm). CT images of a tissue cylinder with seven bone inserts (densities 1.0 to $2.2\;g/{\mathrm{cm}}^{3}$) were simulated to assess material decomposition accuracy. Multiple noisy simulations of an anthropomorphic phantom were performed to generate pixel-by-pixel noise profiles.

**Results:**

PCDs yielded a 15% to 45% improvement in single-line integral SNR for both materials. In CT simulations, similar material decomposition accuracy was achieved, with both EIDs and PCDs slightly underestimating bone density. However, PCDs yield a higher contrast-to-noise ratio and more uniform noise texture than EIDs in virtual monoenergetic images.

**Conclusions:**

We demonstrate the potential for improved MV-kV DE CT imaging using non-spectral PCDs and quantify the degree of improvement in a range of object compositions. This work motivates the experimental assessment of PCDs for megavoltage imaging and the potential clinical translation of PCDs to radiotherapy imaging.

## 1 Introduction

“MV-kV” dual-energy (DE) computed tomography (CT) is a proposed imaging modality with potential application in radiotherapy settings.^1^^–^^3^ Modern radiation therapy systems are already equipped with the hardware necessary for single-shot DE-CT: two x-ray source-detector arrays that rotate in tandem. The first source operates in the megavoltage (MV) energy range. Its primary purpose is radiation therapy treatment, but it can also be detuned to operate with a softer energy spectrum (peak photon counts at ≈1  MeV) appropriate for imaging. The second source operates in the kilovoltage (kV) energy range (80 to 140 kVp) for on-board imaging during radiation therapy. By imaging with both sources simultaneously, one can conveniently realize the benefits of single-shot DE imaging available in diagnostic settings without the need to purchase or install specialized equipment.^4^^–^^6^

MV-kV DE-CT shows promise for improved soft-tissue contrast during routine radiotherapy imaging.^1^ Because images acquired with distinct x-ray energy spectra have distinct contrast levels, DE-CT enables the decomposition of raw image data into two or three basis material images, in which each pixel value corresponds to the density of a chosen material such as bone or tissue.^7^ These material images may in turn be used to generate virtual monoenergetic images (VMIs), synthetic estimates of the images that could be acquired with an ideal single-energy spectrum. VMIs can be generated at low energies with relatively high native soft-tissue contrast and additionally alleviate common single-kV imaging issues such as beam-hardening artifacts. These aspects are highly advantageous when aiming to visualize a tumor embedded in a similarly absorptive soft-tissue background.

In previous work, we demonstrated the ability of MV-kV DE-CT to generate images of the pelvis with higher native contrast than can be achieved with dose-matched single-kV images.^1^ The technique might also be used for metal artifact correction due to the high penetrability of megavoltage-energy photons. However, the limitations of MV imaging can curb the benefits of MV-kV DE-CT in common imaging scenarios. Though fewer MV x-rays are attenuated by the body relative to kV x-rays, they deposit a much greater dose per stopped photon. To deliver the same dose as a kV spectrum, an MV spectrum’s flux must be considerably reduced, resulting in much noisier images. Furthermore, MV detective efficiency is generally much lower than kV efficiency when using currently available x-ray detectors.^8^^–^^10^ When implementing MV-kV DE-CT imaging, the greater noise of the MV data can contaminate the kV data and result in VMIs with an overall lower contrast-to-noise ratio (CNR). Clinical translation of MV-kV DE-CT will likely require a technique that reduces the noise of MV images.

Though image noise might always be decreased by increasing incident flux, this results in a higher imaging dose, which is undesirable in radiotherapy imaging settings. Unlike in diagnostic imaging, a patient must be imaged repeatedly in a relatively short period of time to track their body morphology and tumor progression for precise and accurate treatment planning. The total dose of this required imaging can sum to the scale of a treatment fraction, motivating the search for accessible, high-contrast, low-dose imaging for radiotherapy.^11^ Ideally, the dose in the context of radiotherapy imaging would be equivalent to or lower than that in diagnostic settings to account for the patient’s repeat imaging. A recent literature review reported a diagnostic reference dose level range of 10 to 15 mGy for chest-abdominopelvic CT tumor imaging.^12^ Historically, MV CT for radiotherapy has been limited by the much higher dose it requires for sufficient image quality (IQ).^13^

An appealing avenue for improved MV IQ might be found on the detector side.^3^ The conventionally implemented MV imaging options are energy-integrating detectors (EIDs). Recent advances in MV imaging with EIDs have predominantly focused on improving detective efficiency. Most MV imaging uses flat-panel electronic portal imaging devices (EPIDs) with efficiencies as low as 1% to 2%.^10^ MV CT with these detectors requires a dose on the order of 300 mGy for sufficient IQ.^14^ Novel EPIDs are being developed with improved efficiency in the range of 5% to 20% through a variety of strategies, such as layering of multiple scintillators or development of new scintillating materials.^10^^,^^11^^,^^15^^,^^16^ With these emerging detectors, doses of 40 mGy can yield acceptable soft-tissue contrast.^17^ Modern tomotherapy systems are also capable of relatively high-efficiency MV CT imaging, with a fan-beam xenon gas detector that stops ∼20% of MV photons.^8^^,^^9^^,^^18^^,^^19^ These systems can achieve acceptable soft-tissue contrast with doses as low as 10 mGy.^20^

However, EIDs have intrinsic downsides aside from detective efficiency. EIDs can be designed with various electronic schemes, either direct or indirect conversion. A common EID used in medical imaging is the scintillating detector, which measures x-ray intensity through an indirect conversion to visible light.^21^ The uniting characteristic of EIDs is that they weigh detected photons by their incident energy and sum them into the final signal. This results in low-energy photons, which have greater soft-tissue contrast than high-energy photons, contributing less information to the final image. Although this reduces both kV and MV image contrast, it is especially unfortunate for MV imaging, in which the highest energy photons are weighted a full two orders of magnitude more than the most useful low-energy photons. A second downside of EIDs is that they suffer from electronic noise, which is independent of the unavoidable quantum noise associated with photon counting statistics. Because the flux of an MV treatment beam must be greatly decreased to deliver an acceptably low imaging dose to the patient, detected photon counts are generally much lower, and electronic noise can become a more significant component of the final image. These two effects result in both lower contrast and higher noise, doubly detrimental to CNR.

As an emerging alternative, photon-counting detectors (PCDs) avoid the energy weighting issue of EIDs. Medical imaging PCDs commonly utilize semiconductors that directly convert x-rays into electron-hole pairs.^22^ Incident photons are registered as electric pulses with amplitude proportional to their energy. One can define one or more pulse-height thresholds and register each signal surpassing the threshold as one count, bypassing the issue of energy weighting and also thresholding out the electronic noise background. PCDs face downsides that can degrade spatial and energy resolution, including K-escape, charge sharing, and pulse pile-up.^23^ The severity of these effects depends on the detector materials and geometry.^24^ Correction techniques are being developed to account for these factors and even apply them advantageously; for example, charge-sharing measurements have been used to achieve sub-micron resolution in a PCD for CT imaging.^25^^–^^27^ Further, PCDs have recently debuted in diagnostic CT scanners and show great potential for improving IQ relative to conventional EIDs.^28^

A key promise of PCDs is that, if desired, one can extract spectral information by defining multiple pulse-height thresholds. These “spectral PCDs” enable multi-energy imaging without the historical need for repeat acquisitions, dual source-detector arrays, multi-layer detectors, fast-kVp switching sources, or other specialized equipment.^23^ With a spectral PCD, multi-energy imaging could be implemented on a radiation therapy treatment system using a single MV spectrum. However, the imposition of spectral thresholds significantly reduces the photon counts per energy bin, exacerbating the key challenge of MV CT—the high noise due to low flux at acceptable dose levels. We address the case of single-MV imaging with a spectral PCD in a Supplementary Material.

The benefits of PCDs might still be realized in the context of radiation therapy imaging by MV-kV DE-CT with “non-spectral PCDs.” These detectors essentially operate as a spectral PCD with one very low-energy threshold to remove electronic noise. Even without any energy resolution, non-spectral PCDs have great potential for improving MV imaging by alleviating the high-energy photon up-weighting issue and reducing low-dose image noise—the two key issues of EIDs. To gain multi-energy information with non-spectral PCDs, one requires a traditional dual-source setup, as in MV-kV DE-CT on a radiation therapy treatment system.

From a practical standpoint, the potential implementation of MV-kV DE-CT with non-spectral PCDs depends on the availability of a PCD with sufficient MV counting efficiency. The PCDs implemented in clinical and preclinical CT systems are typically made of cadmium telluride (CdTe), cadmium zinc telluride (CZT), or silicon (Si) semiconductors.^24^ CdTe and CZT have a higher atomic number and therefore can be made relatively thin (mm-scale) for diagnostic imaging. These detectors are likely too thin for MV imaging. Using an estimate based on the linear attenuation coefficient, a 3-mm CdTe slab will stop only 10% of 3-MeV photons. This is better than most commercial EPIDs but only half the reported 20% efficiency needed for sufficiently low-dose MV imaging.^11^ Si PCDs are a more affordable option due to the broader availability of silicon wafers, but for sufficient detective efficiency, they require centimeter-scale thickness due to their lower atomic number.^29^ For this reason, Si detectors have been developed using an “edge-on” approach. Perhaps surprisingly, this could be more advantageous for MV imaging: a 3-cm Si slab is expected to attenuate 20% of 3-MeV photons, which is comparable to the high-efficiency MV EID we modeled. Unfortunately, MV photons will produce larger charge clouds and thus are likely to suffer from more charge sharing. This would be especially prominent in Si detectors, where there is a greater proportion of Compton events contributing to overall photon attenuation.^29^ Although these PCDs may offer sufficient MV efficiency, they may suffer losses in spatial resolution. In an emerging application, some work has explored novel edge-on CZT detectors for positron emission tomography imaging.^30^^–^^32^ These detectors are especially promising for MV imaging, as a 3-cm CdTe slab can stop 50% of 3-MeV photons. The drawback of edge-on CZT detectors relative to Si is likely manufacturing cost. These available and emerging diagnostic CT PCDs show promise for MV imaging and indicate the feasibility of MV-kV DE-CT.

The purpose of this work was to explore the potential utility of non-spectral PCDs in the context of MV-kV DE-CT imaging. The advancing clinical implementation of PCDs makes this topic of particular interest for MV imaging, which faces unique challenges relative to diagnostic kV imaging. Yet, there has been limited investigation of PCDs beyond the diagnostic CT imaging energy range, with tube potentials generally less than 150 kV. We aim to compare IQ achieved with non-spectral PCDs to that achieved with EIDs used for MV imaging on advanced radiotherapy treatment systems and to quantify the degree of potential improvement.

## 2 Methods

We implemented three approaches for comparing MV-kV IQ achieved with non-spectral photon-counting and EID models. Spectral PCDs are discussed separately in the Supplementary Material.

**Fig. 1:**
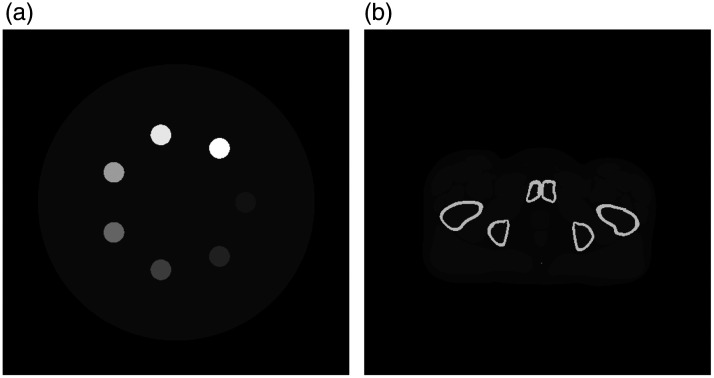
Two phantoms used for the CT simulations. (a) An IQ phantom of tissue with seven bone inserts of varying densities. (b) A section of the XCAT phantom. Pixel values are in HU evaluated at 80 keV and shown at a window level of 0 and width of 1000. Each image field-of-view is 50 cm.

A single-line integral toy model was used to assess a theoretical limit on achievable IQ and to optimize dose allocation between the MV and kV spectra (Sec. 2.2). Simulated MV-kV DE-CT images of an IQ phantom were used to assess material decomposition accuracy with the two detector models (Sec. 2.3). MV-kV DE-CT images of an anthropomorphic phantom were also simulated for qualitative noise analysis (Sec. 2.3). The two phantoms used for the CT simulations are shown in Fig. 1. We first describe the materials and parameters common to these approaches (Sec. 2.1).

### 2.1 Materials

The modeled MV-kV DE system utilized a detuned MV x-ray spectrum and 80-kVp spectrum typical for diagnostic imaging. This MV source is based on a typical 6 MV radiotherapy treatment beam with its spectrum softened such that the average photon energy is below 3 MeV and the maximum flux is at ∼1  MeV.^33^^,^^34^ The detuned beam is achievable on existing treatment systems and more favorable for MV imaging by mitigating some drawbacks of high-energy x-rays, but it would not be used for radiotherapy treatment.

Both EID and PCD models used the same energy-dependent detective efficiency function η(E). This allowed us to isolate the effect of the different photon weighting schemes of the two detector models, which is of particular interest in the high-energy case of MV imaging. The detective efficiency function was modeled based on existing xenon gas EIDs with relatively high detective quantum efficiency in the MV energy range. The function is near unity in the keV energy range and decreases to 20% at energies up to 6 MeV.^1^ Such detectors are implemented in existing tomotherapy systems (Accuray Inc., Sunnyvale, CA, United States) for fan-beam MV imaging.^18^^,^^19^ For our theoretical comparison, we applied this same efficiency function to the PCD model, though such detectors do not exist in reality. Other high-efficiency MV detector options are also emerging, for example, utilizing stacks of EPIDs as EIDs or edge-on PCDs.^11^^,^^29^^–^^32^

**Fig. 2:**
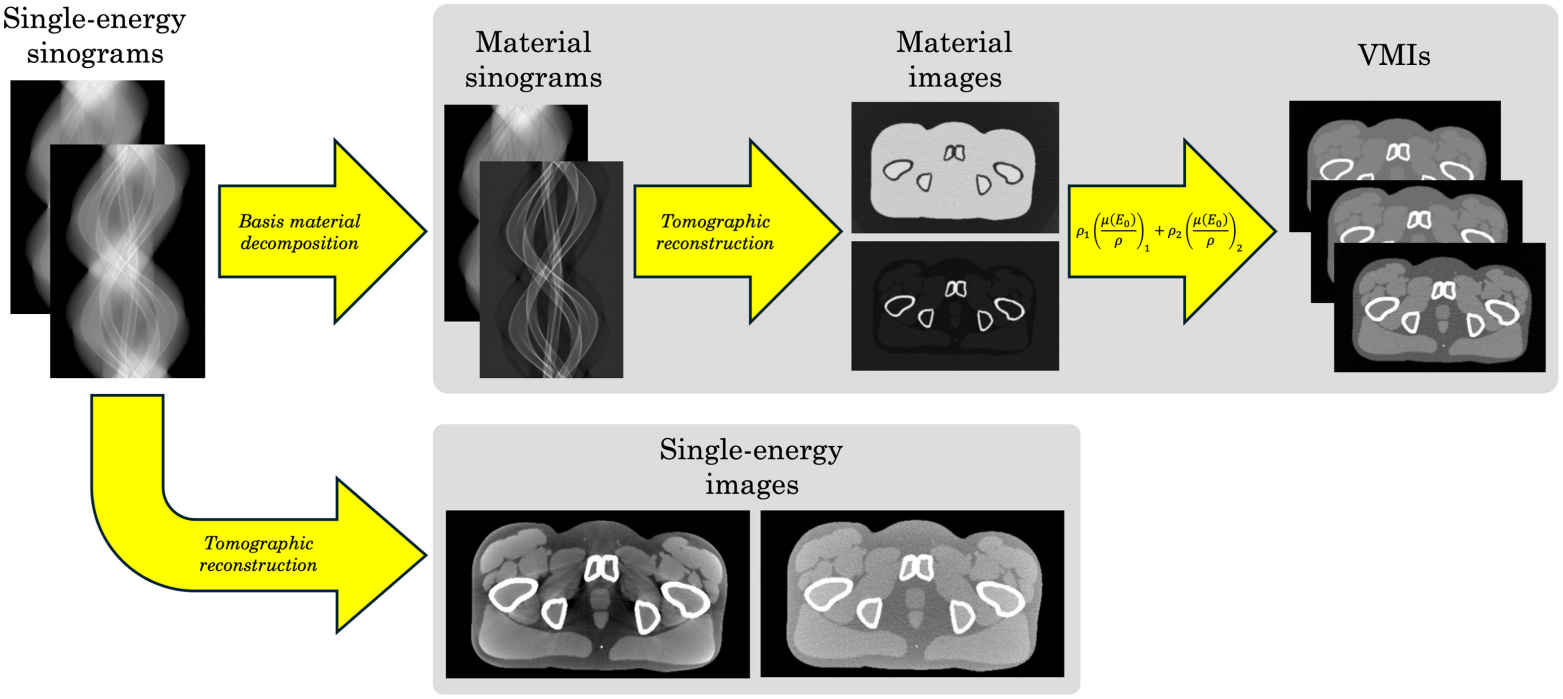
Flowchart summarizing the MV-kV simulation procedure. Single-energy MV and kV sinograms are simulated independently and then recombined for DE-CT (top row): sinogram-domain material decomposition is performed, then material images are reconstructed, and VMIs are generated using Eq. (1). Alternatively, the single-energy sinograms can be directly reconstructed (bottom row): the single-kV image (left) suffers from severe beam hardening, whereas the single-MV image (right) has poor contrast and high noise.

The raytracing CT simulation utilized a 55-cm source-to-isocenter distance and a 100-cm source-to-detector distance. The fan-beam detector had 800 channels and a total fan angle of 50 deg. For all simulations, the two input spectra magnitudes were rescaled such that they would deliver a total dose of 10 mGy to the center of a 40-cm water cylinder.^1^ A total of 1200 projection views were acquired over a 360-deg rotation. For each DE acquisition, sinogram-domain material decomposition into tissue and bone basis materials was performed using a Gauss-Newton algorithm.^35^ Images were reconstructed using fan-beam filtered back-projection with a matrix size of 512, field of view of 50 cm, and ramp filter with cutoff frequency at 80% of the Nyquist limit. VMIs were generated at a continuum of energies ($E_{0}=20-120\;\mathrm{keV}$) as a linear combination of the basis material images $\rho_{j}$:

$$\mathrm{VMI}(E_{0})=\rho_{1}{\left(\frac{\mu (E_{0})}{\rho }\right)}_{1}+\rho_{2}{\left(\frac{\mu (E_{0})}{\rho }\right)}_{2},$$ (1)

where the weights $(\mu (E_{0})/\rho {)}_{j}$ are the known mass attenuation coefficients of basis material j at energy $E_{0}$. The MV-kV DE-CT simulation workflow is summarized as a flowchart in Fig. 2, which includes example images demonstrating how VMIs can alleviate single-energy image artifacts.

### 2.2 Single-line Integral

We implement an estimation theory approach for computing the Cramèr-Rao lower bound (CRLB) on variance in the context of basis material decomposition along a single ray through two materials, summarized here.^36^ We consider two polychromatic x-ray spectra $I_{i}(E)$ (i=1,2) incident on an object comprising two basis materials, each with mass thickness $A_{j}=\rho_{j}\times t_{j}$ (j=1,2). The signal detected using a single spectrum $\lambda_{i}$ can generally be described as

$$\lambda_{i}=\int_{E_{\min}}^{E_{\max}}I_{i}(E)\exp[-\underset{j}{\sum }\mu_{j}(E)t_{j}]\eta (E)D(E)dE,$$ (2)

where $\mu_{j}$ is the linear attenuation coefficient of basis material j, η(E) is the detective efficiency, and D(E) is the detector response function. The signal model and a defined noise model are used to construct the log-likelihood function and, consequently, the Fisher information matrix F. Taking each basis material mass thickness as the parameter of interest, the CRLB $\sigma_{A_{j}}^{2}$ is then computed by inverting the Fisher matrix,

$$\sigma_{A_{j}}^{2}=F_{jj}^{-1}.$$ (3)

Just as the CRLB represents the lower bound on variance for an unbiased estimator, a theoretical upper bound on achievable IQ can be defined as the unitless value $A_{j}/\sigma_{A_{j}}$. Interpreting the true mass thickness as the ideal signal, we refer to this parameter as the single-line integral model signal-to-noise ratio (SNR) for each basis material.

We previously applied this method for comparing MV-kV DE imaging with traditional diagnostic (kV-kV) DE imaging using EIDs only and neglecting electronic noise.^1^^,^^2^ However, the detector response function is distinct for integrating measurements [D(E)=E] and counting measurements [D(E)=1], resulting in different functional forms of the CRLB for either detector model. Furthermore, one major advantage of PCDs is their potential to eliminate electronic noise by applying some minimum pulse-height threshold greater than the noisy background. We assess the utility of this strategy by extending the EID CRLB model to include electronic noise by adding a factor of $\sigma_{e}\times {\overset{¯}{E}}_{i}$ to the EID noise model, where $\sigma_{e}$ is the standard deviation in counts due to the electronic noise and ${\overset{¯}{E}}_{i}$ is the intensity-weighted average energy of spectrum $I_{i}$. We chose $\sigma_{e}=10$ photons. By contrast, the PCD model used $\sigma_{e}=0$, simulating a low pulse-height threshold that eliminates background noise but does not yield other multi-energy information.

We computed ${\mathrm{SNR}}_{j}$ for a range of tissue thicknesses ($t_{1}=10\text{–}50\;\mathrm{cm}$, $\rho_{1}=1.06\;g/{\mathrm{cm}}^{3}$) and bone thicknesses ($t_{2}=0.1\text{–}10\;\mathrm{cm}$, $\rho_{2}=1.85\;g/{\mathrm{cm}}^{3}$). We also considered the effect of dose allocation between the MV and kV spectra. The magnitude of each spectrum was initially scaled such that the total dose delivered by a single polychromatic ray to the center of a 40-cm water cylinder would be 1  μGy.^1^ For a CT scan with 1000 views, this corresponds to a 1-mGy dose. To ensure the total dose remained fixed for each acquisition, the magnitude of the MV spectrum was rescaled by a factor r (ranging from 0.01 to 0.99), and the kV spectrum was rescaled by the remaining 1−r. We compared the dose-optimized SNRs achieved with either an EID or non-spectral PCD model, the dose allocation factor r needed to achieve this optimal SNR, and the effect of basis material thickness.

The resulting SNRs are scalable to other dose levels. For the PCD case, which has a pure Poisson counting noise model, noise is proportional to the inverse square root of flux, so SNR increases with the square root of dose per ray [μGy] at any dose allocation factor. Although this square root rule-of-thumb is not exact for the EID case due to photon energy weighting and electronic noise, these nonidealities are mainly relevant at very low doses, so the rule is still widely applied.^37^^–^^41^

### 2.3 CT Simulations

As an extension of the single-line integral model, we simulated MV-kV DE-CT acquisitions of two phantoms. To assess basis material decomposition with the two detector schemes, we created a computational IQ phantom comprising a 40-cm tissue cylinder with seven 3-cm bone inserts with densities varying from 1.0 to $2.2\;g/{\mathrm{cm}}^{3}$. Twenty MV-kV DE-CT acquisitions were simulated using both EIDs and non-spectral PCDs as described above, providing several noisy realizations for statistical analysis. In the bone basis material image, the mean pixel value in each insert was measured and compared with the known ground truth. CNR measurements were taken in the region of each bone insert, defined as

$${\mathrm{CNR}}_{\rho }(E_{0})=\frac{\left|m_{\rho }(E_{0})-m_{bg}(E_{0})\right|}{\sigma_{bg}(E_{0})}$$ (4)

where the subscript ρ indicates the masked bone insert at the density of interest, the subscript bg indicates the masked tissue background, m represents the mean, σ represents the standard deviation, and $E_{0}$ indicates the energy of the VMI used for the measurements.

For qualitative analysis of image noise profiles using either detector scheme, we simulated MV-kV DE-CT acquisitions of an extended cardiac torso (XCAT) phantom.^42^ We generated twenty noisy CT images using both detector models and synthesized the basis material images into VMIs using Eq. (1). Noise profile images were generated by taking the pixel-by-pixel standard deviation over the multiple noisy realizations.

**Fig. 3:**
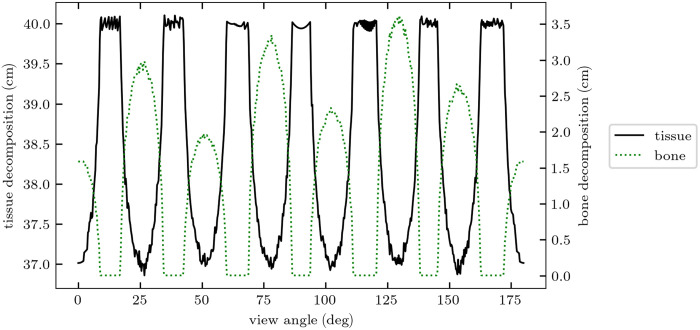
Basis material thickness line profiles through the IQ phantom used for approximate dose allocation optimization. Tissue thickness ranged between 37 and 40 cm, and bone thickness ranged from 1.5- to 3.5-cm equivalent for the seven different density inserts.

**Fig. 4:**
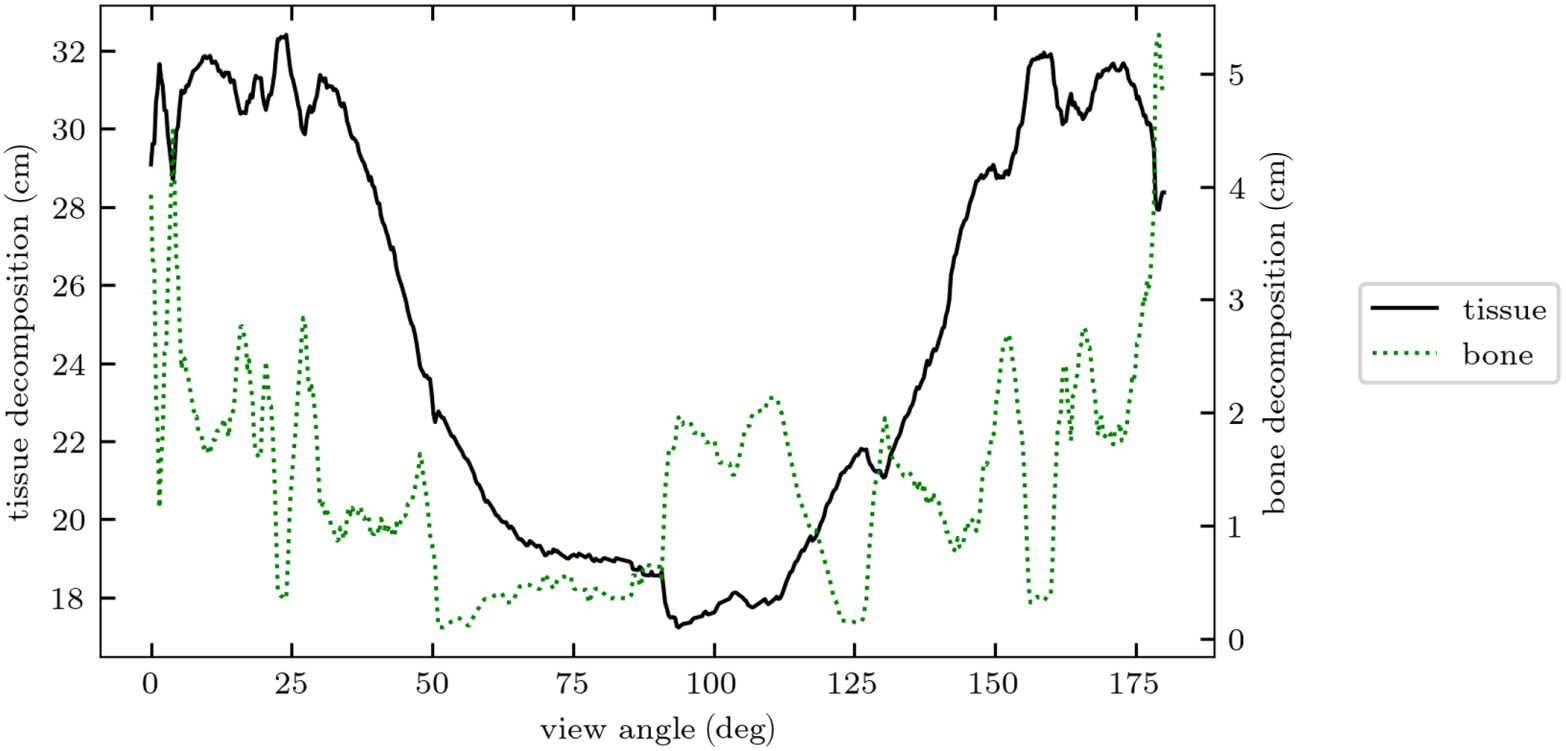
Basis material thickness line profiles through the XCAT phantom used for approximate dose allocation optimization. Tissue thickness ranged between 18 and 32 cm, and bone thickness varied up to 5 cm.

For both phantoms, the total DE-CT dose was fixed at 10 mGy. The dose allocation between the MV and kV spectra was approximately optimized using the results of the single-line integral. An initial 180-deg noiseless simulation with just one central detector channel was conducted to yield basis material thickness line profiles through the phantom, and a single-dose allocation factor r was chosen depending on the varying material thickness pairs over the several projection view angles. The thickness line profiles for the two phantoms are shown in Figs. 3 and 4. Though this method assumed *a priori* knowledge of the phantom composition, it was highly approximate. Similar if not better methods can likely be implemented in clinical scenarios using anterior-posterior and lateral scout scans, as is commonly used for tube current modulation. Based on these thickness profiles, we utilized 85% and 90% doses allocated to the MV spectrum for the IQ and XCAT simulations, respectively.

## 3 Results

### 3.1 Single-line Integral

**Fig. 5:**
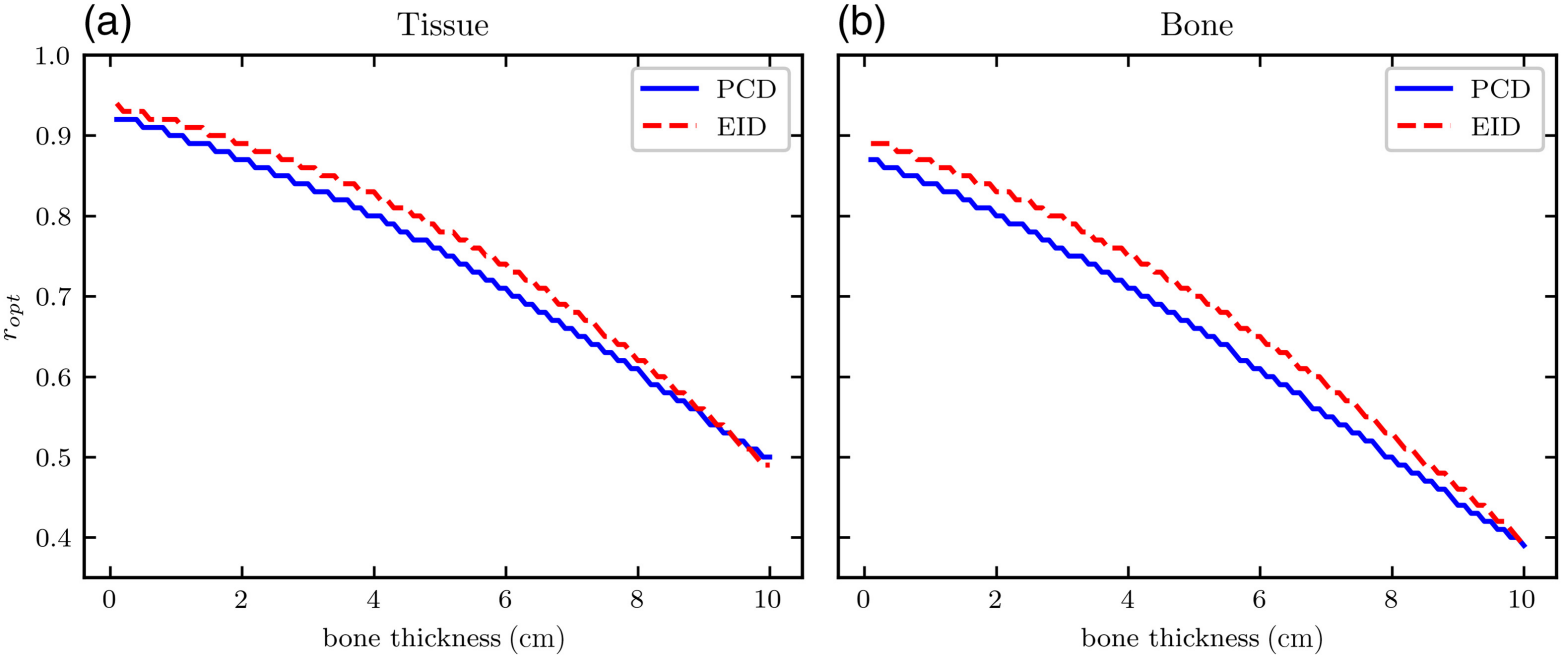
Dose allocation to the MV spectrum maximizing basis material SNR for (a) the tissue and (b) bone as a function of bone thickness with fixed tissue thickness of 40 cm.

Figure 5 shows the dose allocation factor $r_{\mathrm{opt}}$ that optimizes basis material SNR using 40-cm tissue and a variety of bone thicknesses. Relative to the EID SNR, the PCD SNR is optimized with a slightly lower dose allocated to the MV spectrum, though the difference decreases as bone thickness increases. The optimal dose allocation is distinct for the bone and tissue, with bone generally having a slightly lower $r_{\mathrm{opt}}$ than the tissue. The largest changes in $r_{\mathrm{opt}}$ occur with respect to bone thickness: small bone thicknesses (<1  cm) are best with nearly 90% dose allocated to the MV spectrum, whereas higher bone thicknesses are optimized with a more equal 50% dose to the MV spectrum. Based on this, we infer the optimal dose allocation for a given image acquisition depends primarily on object composition, with additional but less pronounced dependence on the material of interest (tissue or bone) and detector (EID or PCD).

**Fig. 6:**
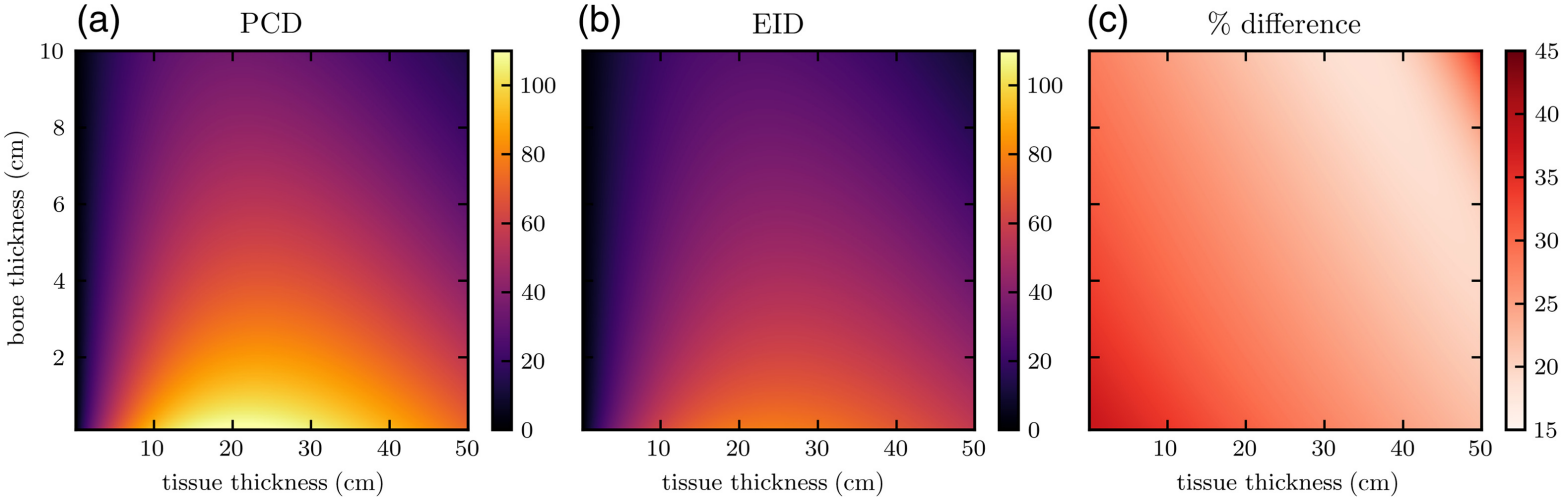
Dose-optimized tissue SNR using (a) PCD and (b) EID models for a variety of basis material thicknesses. Panel (c) shows the percent difference.

**Fig. 7:**
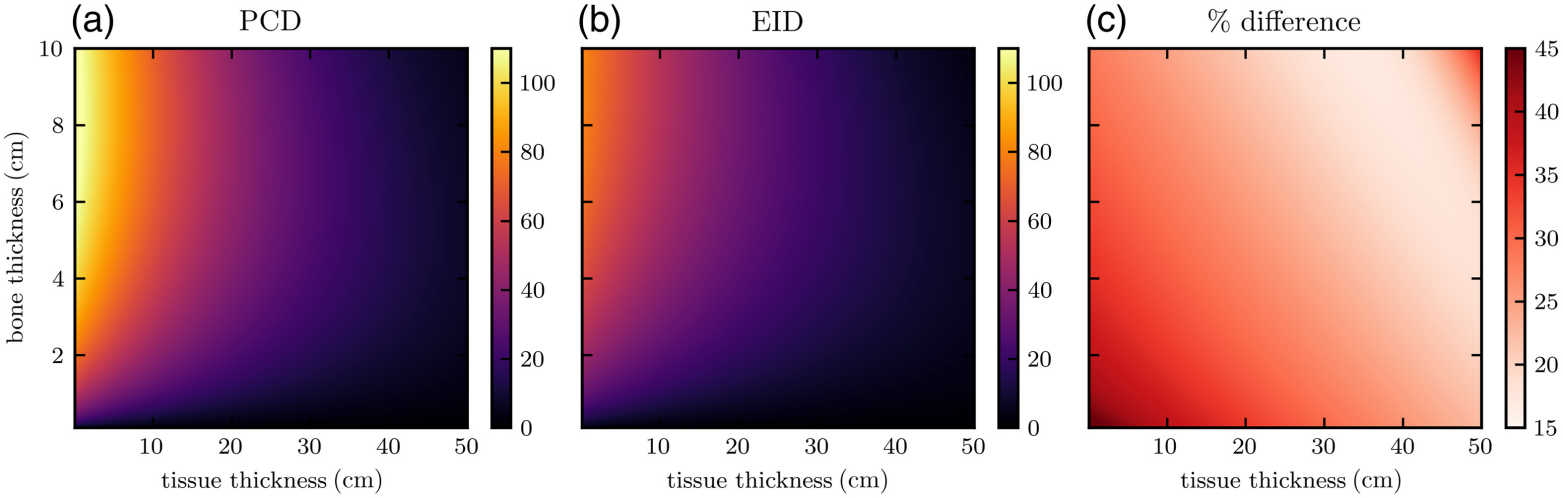
Dose-optimized bone SNR using (a) PCD and (b) EID models for a variety of basis material thicknesses. Panel (c) shows the percent difference.

Figures 6 and 7 show heatmaps of dose-optimized basis material SNR at all bone and tissue thickness pairs assessed. Across all material compositions, PCDs produce between 15 and 45% improvement in SNR. The greatest percent improvements are seen for very thin objects, which also have the lowest overall SNRs due to their lower attenuation. The optimal dose factors that generated the maximum SNRs shown in these heatmaps were used to inform the approximate dose allocation optimization of the CT scans. For example, Fig. 5 shows $r_{\mathrm{opt}}$ as a function of bone thickness for 40-cm tissue. For each phantom, characteristic average tissue thickness and non-zero bone thickness were approximated from the angle-dependent thickness profiles (Figs. 3 and 4). Average non-zero bone thickness was generally low (≈2  cm), where the $r_{\mathrm{opt}}(t_{\text{bone}})$ curve is slowly varying (Fig. 5) and only 2% to 3% different for EIDs and PCDs. From this, we selected the constant values of $r_{\mathrm{opt}}=0.85$ and 0.90 for the IQ phantom and XCAT phantom, respectively.

### 3.2 CT Simulations

**Fig. 8:**
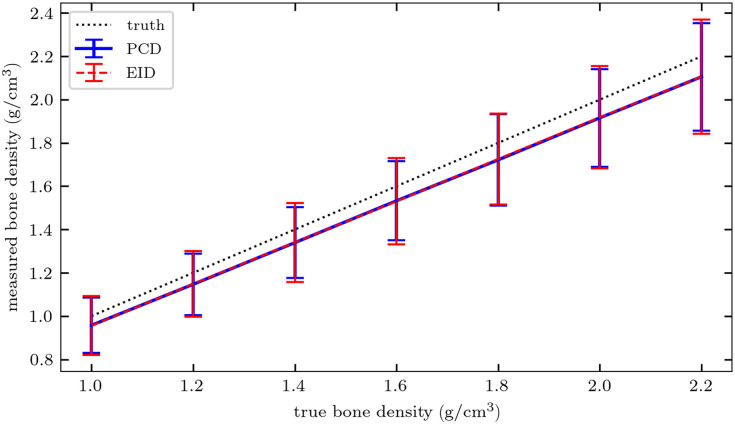
Measured versus true bone density in the seven inserts of the IQ phantom. Error bars represent k=1 standard deviation over all noisy simulations.

**Fig. 9:**
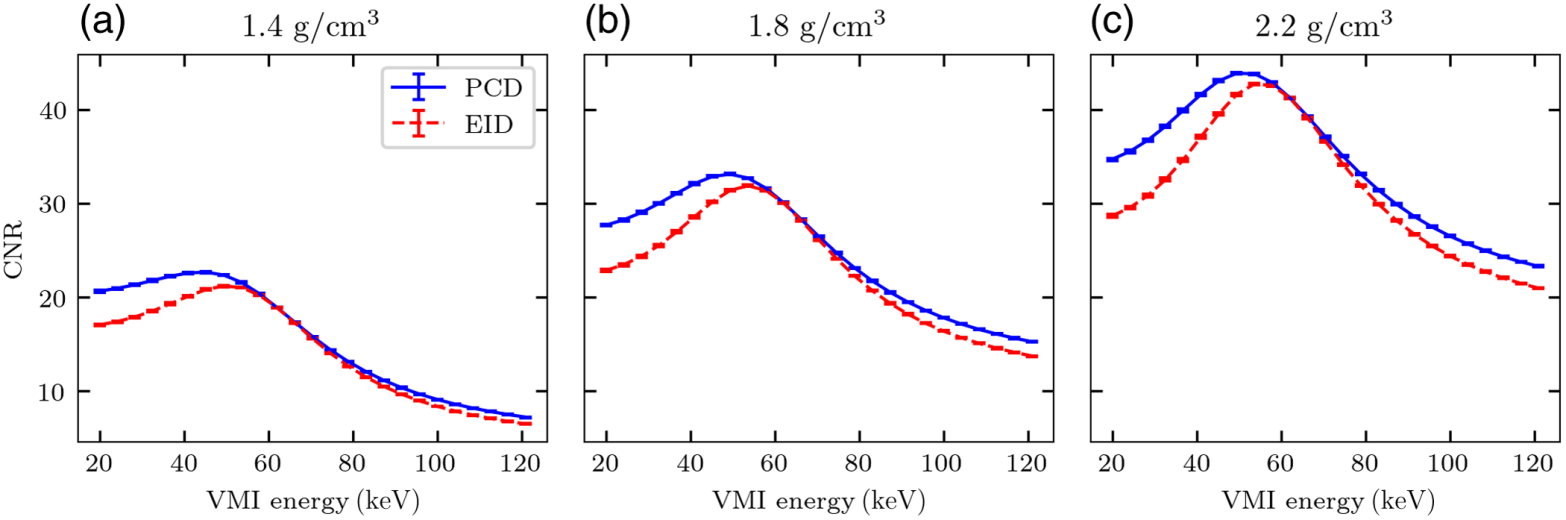
CNR measured in three bone inserts with density (a) $1.4\;g/{\mathrm{cm}}^{3}$, (b) $1.8\;g/{\mathrm{cm}}^{3}$, or (c) $2.2\;g/{\mathrm{cm}}^{3}$ as a function of VMI energy. The error bars represent k=2 standard deviations over all noisy simulations.

To assess basis material decomposition accuracy for the two detector schemes, Fig. 8 shows bone basis material measurements in the seven IQ phantom inserts. The measurements are similar using either PCD or EID at all densities, and both detector schemes result in a slight underestimation of true bone density. However, the basis material images of PCDs and EIDs produce distinct noise levels in VMIs, as illustrated in Fig. 9, which shows CNR in three bone density inserts at a continuum of monoenergies. PCDs consistently yield higher VMI CNR than EIDs at all monoenergies, with peak CNR generally occurring at a slightly lower monoenergy. The improvement in CNR with PCDs is especially pronounced at lower (<50  keV) VMI energies.

**Fig. 10:**
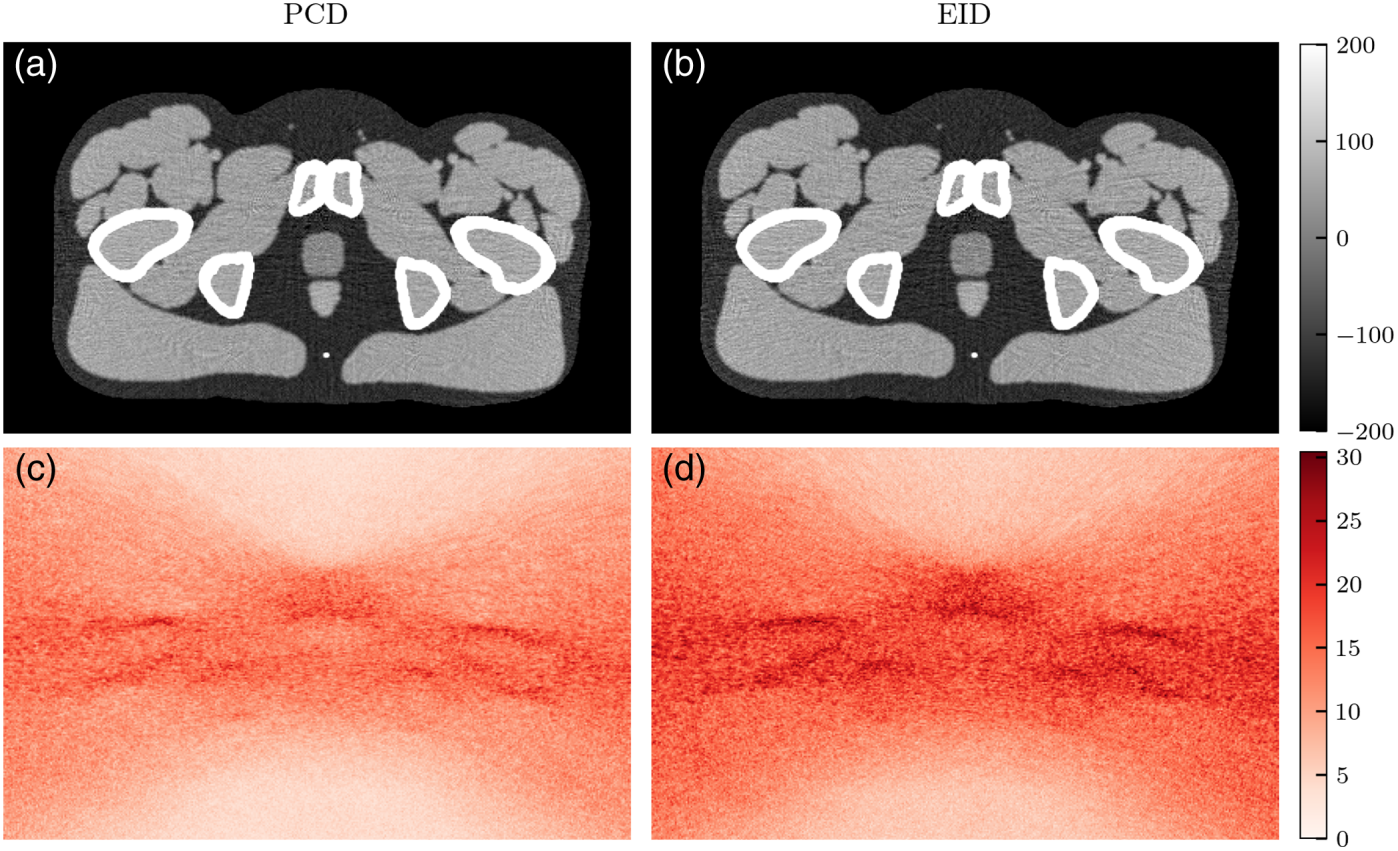
(a)–(b) 50-keV VMIs and (c)–(d) their noise profiles simulated with either PCD (a), (c) or EID (b), (d) schemes. Pixel units are HU. The noise profile is concentrated around the bone regions.

**Fig. 11:**
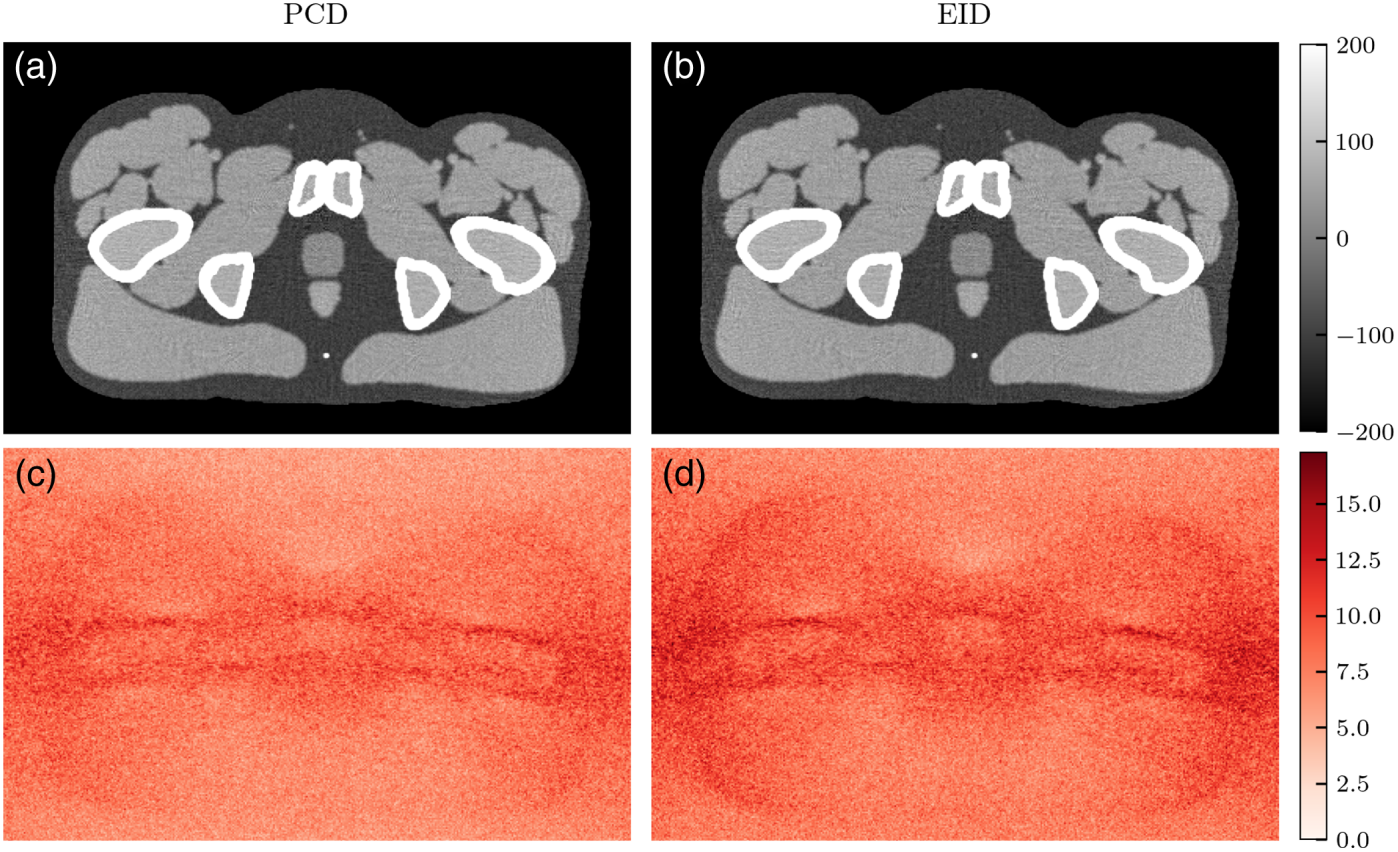
(a)–(b) 60-keV VMIs and (c)–(d) their noise profiles simulated with either PCD (a), (c) or EID (b), (d) schemes. Pixel units are HU. The noise profile is distributed around the bones and lateral edges of the phantom.

**Fig. 12:**
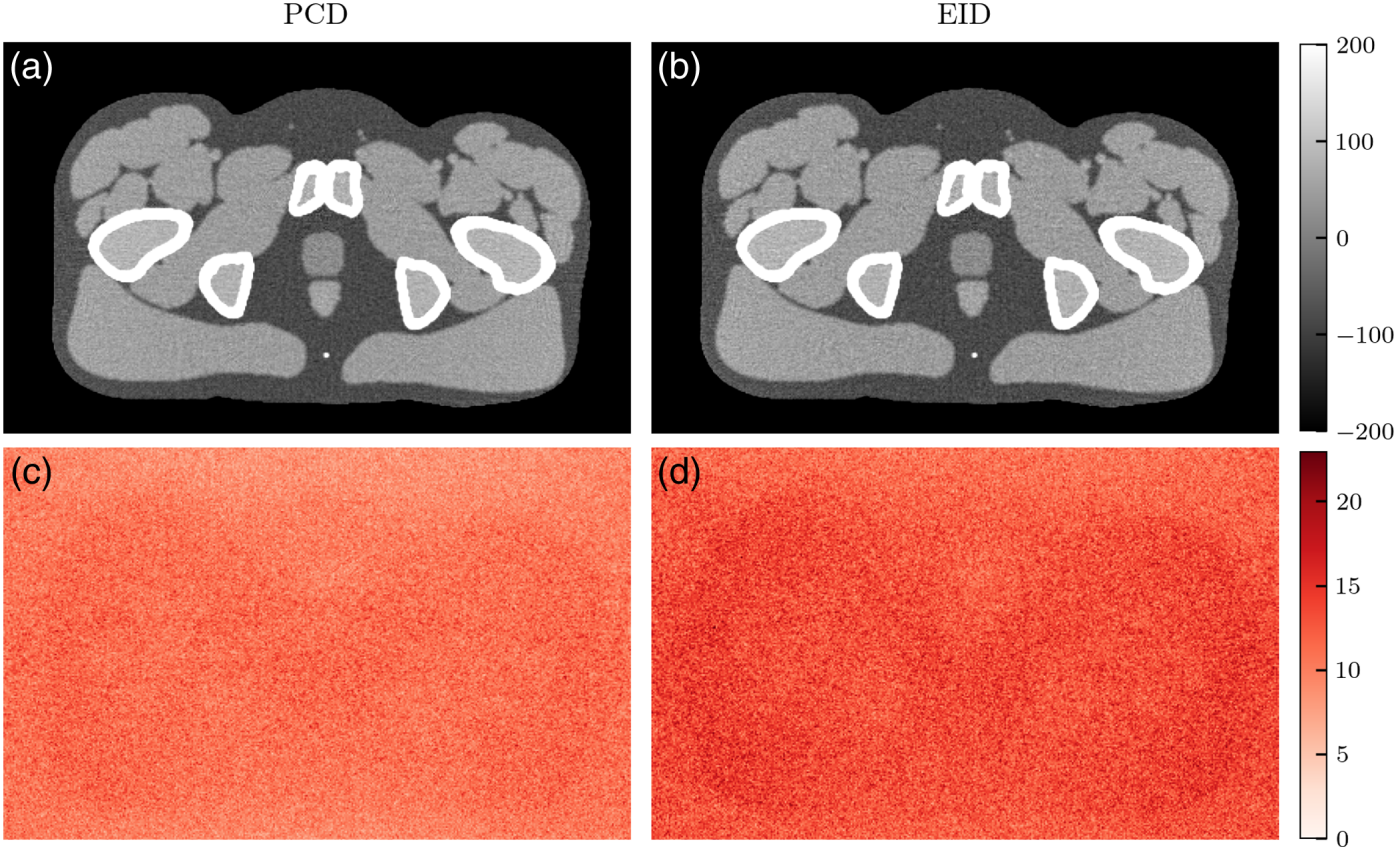
(a)–(b) 70-keV VMIs and (c)–(d) their noise profiles simulated with either PCD (a), (c) or EID (b), (d) schemes. Pixel units are HU. The noise profile has a more uniform texture across tissue regions.

Figures 10–12 show VMIs and their corresponding noise profiles using either the PCD or EID model. Broadly, EIDs result in noisier images. In the lower energy VMIs (50 keV), the noise profile is concentrated around bony regions and in streaks between bones. Although this is apparent for both detector models, it is more pronounced with EIDs. This results in a less isotropic noise texture in the EID images. As VMI energy increases to 60 and 70 keV (Figs. 11 and 12), the noise texture shifts to be more isotropic across the tissue regions of the phantom. Although the EID images still have greater noise magnitude than the PCD images, the two noise textures appear to be more similar.

## 4 Discussion

MV-kV DE-CT imaging is a proposed modality that could be implemented in existing radiotherapy treatment systems, possibly bringing the advantages of multi-energy imaging to routine radiotherapy imaging over the course of a patient’s cancer treatment.

In this work, we explored the implications of exchanging the EIDs in a previously studied MV-kV DE-CT system with non-spectral PCDs of identical counting efficiency.^1^ This allowed us to assess the effect of the energy-weighting detection scheme and electronic noise. We expected the energy-weighting effect of EIDs is particularly detrimental for MV imaging due to the multiple order-of-magnitude increase in weighting for photons carrying the least contrast information. A 3-MeV photon would be weighted 100× more than a 30  keV photon. Furthermore, because the MV spectrum flux must be greatly reduced relative to the kV spectrum to reach acceptable dose levels, we expected the effect of a constant electronic noise background may be more pronounced. Our findings corroborate these expectations.

In the single-line integral model, for each basis material, dose-optimized SNR initially increases with thickness as the true mass thickness ρ×t (numerator) increases. Each SNR eventually peaks due to increases in the CRLB (denominator), likely due to a reduction in detected photon counts as they are attenuated by the object. This general relationship is true for the two detector models. Comparing PCD SNRs to EID SNRs, we observe a saddle surface shape in the percent improvement heatmap for all material thickness pairs (panel (c) of Figs. 6 and 7). The greatest improvement (approx. 45%) in SNR was seen at low thicknesses, where the signal is the weakest but the electronic noise background is less significant relative to the large number of detected photons. PCDs yield a smaller but still observable (approx. 15%) improvement at more moderate thickness pairs. At very high thicknesses, the percent improvement increases to near 30%, likely due to the high attenuation of the incident beams resulting in a more dominant electronic noise component in the EID signal.

The dose optimization factor $r_{\mathrm{opt}}$ was slightly different depending on the detector model, and 0 to 5% less of the total dose should be allocated to the MV spectrum when using a PCD. Tables of $r_{\mathrm{opt}}$ values for all material thickness pairs were used to choose the dose allocations of 0.85 and 0.90 for the IQ phantom and XCAT phantom CT simulations, respectively. More specifically, $r_{\mathrm{opt}}$ varies depending on the specific thickness pair of each view angle as in Figs. 3 and 4. In a separate experiment, we simulated CT scans with $r_{\mathrm{opt}}$ modulated as a function of view angle according to the thickness profiles for each phantom and detector type. We found that CNR did not significantly improve relative to the CNRs for the constant values of 0.85 and 0.90, indicating that these constant $r_{\mathrm{opt}}$ choices are sufficient for this study. The scope of this paper focuses on fairly comparing EID and PCD performance, not necessarily optimizing performance as a function of dose allocation. This subject might be further investigated in future work.

In the MV-kV DE-CT simulations, the Gauss-Newton material decomposition method implemented had similar accuracy for either EID or PCD detector model.^35^ By combining the two basis material images into a VMI, greater CNR can be achieved at a range of monoenergies. As expected, CNR increases with bone density due to the greater attenuation of the signal relative to the background, and the monoenergy maximizing CNR increases with bone density due to beam hardening. For each individual bone density, PCD CNR was greater than EID CNR at all VMI energies examined (20 to 120 keV). The peak PCD CNR occurred at a slightly lower monoenergy than the peak EID CNR. This might be attributed to the improvement in PCD basis material image noise. In principle, the two material images’ noise is correlated such that when they are recombined using Eq. (1), the resulting VMI will have less noise. This depends on the energy. Because the mass attenuation coefficients in the VMI calculation decrease as energy increases, low-energy VMIs can amplify the noise in the material images. This effect is not as severe for the less noisy PCD images, so the peak CNR is at a lower monoenergy with higher native contrast. The greatest CNR improvements were observed at low monoenergies (<50  keV), whereas only a slight improvement was seen after peak PCD CNR. For a more clinically relevant assessment, we also simulated MV-kV VMIs of an anthropomorphic phantom. Qualitatively, for both detectors, lower monoenergies produced higher soft-tissue contrast but greater noise, especially around regions with bone and along the lateral patient axis (Fig. 10). The noise texture and magnitude are more prominent in the EID images. For both detector models, soft-tissue contrast decreased and noise texture became more uniform at higher monoenergies, but the noise magnitude was still higher using the EID (Figs. 11 and 12). The greatest improvements in IQ with the PCD appear to be due to improvements in noise magnitude and texture, whereas contrast is subjectively more similar.

The results of this computational assessment of non-spectral PCDs for MV-kV DE-CT motivate future experimental work. The CT simulation study provides an initial estimate of the noise reduction benefits achievable with PCDs. An experimental study would more realistically include other factors such as photon scatter, spatial resolution, and available PCDs. These results could be compared with simulated findings for a better understanding of the clinical translatability of our results. Simulations and estimation theory calculations allow us to conveniently explore a range of parameters relevant to radiotherapy imaging scenarios, complementing these experimental investigations. These computational methods could also be used to optimize image acquisition parameters to ensure fair comparison of different detector models.

## 5 Conclusion

PCDs show promise for improving MV-kV DE CT imaging by improving MV image noise characteristics. In terms of single-line integral basis material SNR, we found that non-spectral PCDs yielded a 15% to 45% improvement over EIDs. In CT simulations, material decomposition accuracy was similar for both detectors, whereas non-spectral PCDs resulted in higher CNR and more uniform noise texture in VMIs. Relative to existing high-efficiency MV EIDs, similar or better MV detective efficiency might be achieved by emerging diagnostic PCDs if utilizing an edge-on setup. This computational study motivates the experimental assessment of PCDs for MV imaging and the potential clinical translation of PCDs for MV-kV DE-CT imaging on radiotherapy systems.

## Supplementary Material



## Data Availability

The code used to generate the data in this study can be freely accessed through GitHub at https://github.com/gjadick/dex-single-ray for the single-line integral toy model and https://github.com/gjadick/dex-ct-sim for the fan-beam CT simulation and basis material decomposition.
