# Supplementary material for: Utility of photon-counting detectors for MV-kV dual-energy computed tomography imaging
Source: J Med Imaging (Bellingham). 2024 Dec 26;11(Suppl 1):S12811. doi: 10.1117/1.JMI.11.S1.S12811 (PMC11670364; doi:10.1117/1.JMI.11.S1.S12811)
Supplement: Supplementary file 1 [file JMI_011_S12811_SD001.pdf]

Spectral detectors with a single-MV spectrum

An additional area of interest is spectral imaging with a single MV x-ray source. While the main text focuses on non-spectral detectors, PCDs can also possess the spectral capability to resolve the energy of incident photons. In addition to simply counting each incident photon, equally weighting each regardless of energy, a spectral detector sorts photons into energy "bins" that span predefined ranges of pulse heights. This detection scheme yields multi-energy data from a single x-ray spectrum.[22–24] Such single-shot spectral imaging can similarly be achieved with EIDs using multi-layer detector, where each layer provides a measurement of its absorbed energy.[16,43] Spectral imaging enables the possibility of material decomposition without need for a dual-energy acquisition.[44]

This subject has traditionally been explored in the context of diagnostic kV spectra. If feasible with MV spectra, the technique might be a great advantage for radiotherapy imaging on systems lacking a kV source entirely. Radiotherapy systems must have some on-board imaging device for patient positioning verification at the time of treatment. Since MV localizers can be sufficient for this purpose and there are practical costs associated with an additional kV x-ray source, not all systems are equipped with dual MV-kV sources.[45] Generally, kV images are more desirable for their higher contrast and lower noise relative to MV images acquired with the same dose. Energy-discriminating PCDs could, in theory, be used with a MV beam for on-board, single-shot spectral imaging and subsequent reconstruction of higher contrast images without need for a kV x-ray source.

This might offer advantages over single-MV imaging. Though the imposition of spectral thresholds would reduce the photon counts of each detected energy bin, this might be alleviated relative to a single-MV acquisition by recombining spectral data in VMIs. Like single-MV imag-

ing, the utility of spectral MV imaging will likely depend on having sufficiently high detective efficiency. It might also benefit from more extensive treatment beam detuning, so that flux could be increased without higher dose and that more low-energy information would be available to complement high-energy information for material decomposition. However, in comparison to MV-kV DE imaging, dose-matched spectral MV imaging will always have a much lower total flux due to the greater dose deposition per attenuated MV photon. As concluded in the main text, MV-kV DE imaging is optimized with most incident flux allocated to the MV beam, depending on object composition. Energy bins in spectral MV imaging could not be dose-optimized due to the fixed shape of the x-ray spectrum. For these reasons, in most scenarios, we do not expect spectral MV imaging to outperform MV-kV DE imaging. In this document, we quantitatively investigate MV imaging with a spectral PCD.

We modeled a spectral PCD with one, idealized energy threshold to sort detected photons into two energy bins. We applied the single-line integral model to probe achievable basis-material SNR as a function of this threshold. The same detective efficiency model as described in Section 2.1 was implemented. The object was modeled as 37-cm of tissue and 3-cm of bone, as in the IQ phantom used for the dual-energy simulations. We considered either a megavoltage spectrum (detunedMV) or a standard diagnostic kilovoltage spectrum (120kV) applied independently in combination with the spectral PCD. Figure S1 shows the shape and magnitude of the spectra, which were scaled to deliver the same 1-μGy dose to the center of a 40-cm water cylinder. As kV photons generally deposit less dose than a MV photon, the magnitude of counts in the kV spectrum is much higher than it is in the MV spectrum.

Figure S2 shows the estimated basis material SNR as a function of spectral threshold for the kV and MV acquisitions. The location of the peak of each curve indicates the optimal spectral

threshold for imaging this material pair. The peak SNRs achieved with the kV spectrum are far higher than those achieved with the MV spectrum, which reaches a maximum SNR of approximately 1.2 for tissue. For both basis materials, the 120kV SNR is maximized with an ideal spectral threshold of 70 keV. The maximum SNRs are 153 for bone and 31 for tissue, which are greater than the non-spectral, dose-optimized MV-kV SNRs shown in Fig. 6 and 7 (67 and 14, respectively). This likely owes to the large difference in total counts between the kV and MV spectra, the order-of-magnitude difference in the modeled detective efficiency function at kV and MV energy ranges, and lower native contrast at higher x-ray energies. The difference may also be attributed to the material thicknesses that were assessed (37 cm of tissue and 3 cm of bone). At greater object thicknesses, spectral MV or non-spectral MV-kV systems have greater potential to outperform single-kV systems.[1] Furthermore, this initial simple experiment utilized a highly idealized spectral response, granting a large advantage to the kV spectral PCD relative to the MV-kV non-spectral PCD single-line SNRs. In generating the spectral signals, we assumed the electrical signal corresponding to each stopped photon is perfectly proportional to its energy. In reality, the amplitude recorded by the detector is distorted by K-fluorescence, Compton scatter, and charge sharing.[24] These non-idealities are material-specific, with CdTe- and CZT-based PCDs more affected by K-fluorescence and Si-based PCDs measuring more low-energy Compton interactions. A more realistic spectral response might be modeled by a Gaussian photopeak centered at the incident photon energy with a low-energy tail.[46,47]

The primary outcome of this experiment is motivation for our focus on MV-kV dual-energy imaging with a non-spectral PCD rather than MV-only imaging with a spectral detector. In future work, it may be of interest to explore a dual-energy MV-kV system that uses a spectral PCD, which could utilize a more advanced basis material decomposition approach to benefit from more

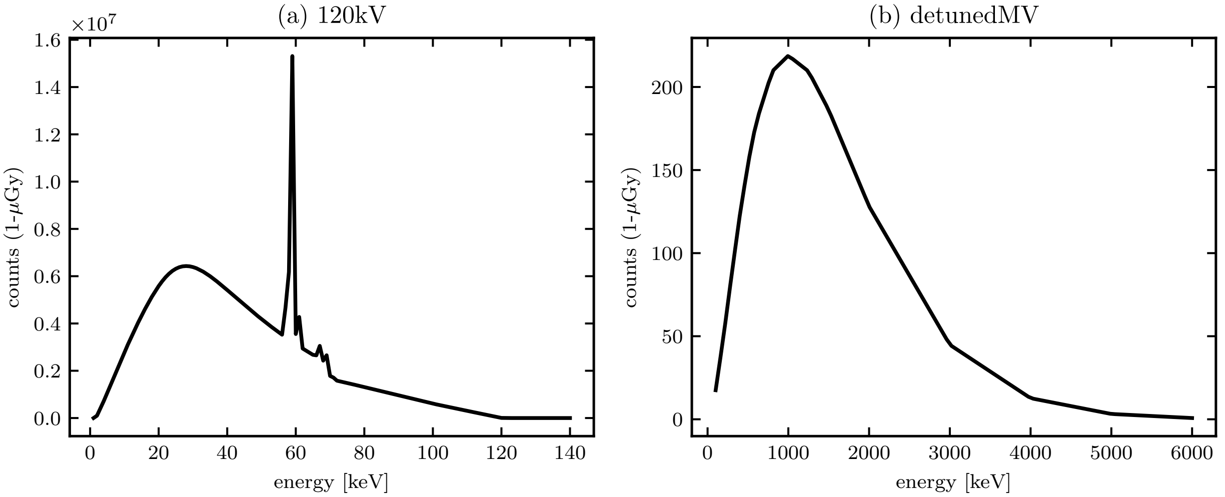

Fig S1 The (a) kilovoltage and (b) megavoltage x-ray spectra with photon counts per 1-keV energy bin scaled to deliver approximately 1-μGy dose to the center of a 40-cm water cylinder.

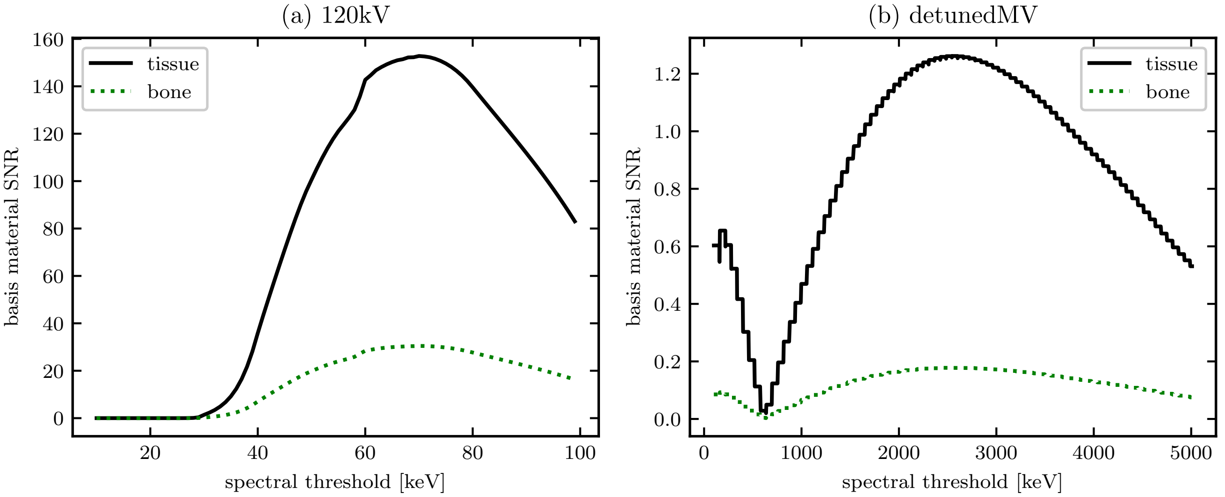

Fig S2 Single-line integral basis material SNR measured as a function of spectral threshold imposed on the (a) kilovoltage and (b) megavoltage spectra for an object composed of 37-cm tissue and 3-cm bone.

than two measurements. Work focusing on this subject should take care to implement a realistic PCD spectral response. We expect that megavoltage-only imaging with a spectral PCD will require much higher dose or greater detective efficiency to be of clinical utility. With a non-spectral PCD, a combination of megavoltage and kilovoltage spectra is likely a more feasible approach to material decomposition on a radiotherapy system.
